# Characterization of Total-Phosphorus (TP) Pretreatment Microfluidic Chip Based on a Thermally Enhanced Photocatalyst for Portable Analysis of Eutrophication

**DOI:** 10.3390/s19163452

**Published:** 2019-08-07

**Authors:** Dong Geon Jung, Daewoong Jung, Seong Ho Kong

**Affiliations:** 1 School of Electronics Engineering, Kyungpook National University, Daegu 41566, Korea; 2 AI System Engineering Group, Korea Institute of Industrial Technology (KITECH), Yeongcheon 38822, Korea

**Keywords:** total-phosphorus, pretreatment, eutrophication, photocatalyst, sensor

## Abstract

To minimize conventional total-phosphorus (TP) analysis systems, TP pretreatment microfluidic chip is proposed and characterized in this paper. Phosphorus (P) is one of the most important elements in ecosystem but it causes the eutrophication due to its overdose. TP analysis systems are increasingly receiving attention as a means to prevent eutrophication. Even though conventional TP analysis systems have high accuracy and sensitivity, they are not frequently utilized outside the laboratory because of their bulky size, complicated pretreatment processes, long response times, and high cost. Thus, there is a growing need to develop portable TP analysis systems. The microfluidic chip in this study is proposed with the aim of simplifying and minimizing TP analysis by replacing the conventional pretreatment process with a new method employing a thermally enhanced photocatalytic reaction that can be applied directly to a microfluidic chip of small size. The fabricated TP pretreatment microfluidic chip with thermally enhanced photocatalyst (TiO_2_) was optimized compared to the conventional pretreatment equipment (autoclave). The optimum pretreatment conditions using the proposed chip were pretreatment time of 10 min and temperature of 75 °C. The optimized pretreatment process using the proposed microfluidic chip showed similar performance to the conventional pretreatment method, even with shorter pretreatment time. The shorter pretreatment time and small size are advantages that enable the TP analysis system to be minimized. Therefore, the proposed TP pretreatment microfluidic chip based on thermally enhanced photocatalytic reaction in this study will be utilized to develop a portable TP analysis system.

## 1. Introduction

In recent years, there has been a growing need for the development of a total-phosphorus (TP) analysis system, which is portable, small-sized, inexpensive, and with short analysis time, and related researches are actively conducted. In general, phosphorus (P) is the most important nutrient in living organisms and exists in the form of compounds in combination with other organic/inorganic substances [1,2,3,4,5]. However, as the concentration of phosphorus in the aquatic ecosystem increases, algae proliferation becomes active, and eutrophication of the aquatic ecosystem occurs [6,7,8,9,10,11,12,13,14,15,16,17,18,19,20,21,22,23,24,25,26,27,28,29,30,31,32,33,34,35,36,37,38,39]. Therefore, in order to prevent eutrophication in advance, it is necessary to be able to monitor the concentration of phosphorus in aquatic ecosystem quickly and accurately. However, existing phosphorus concentration monitoring equipment (or system) is very bulky, expensive, and takes a long time to analyze. Due to these disadvantages, it is very difficult to analyze phosphorus concentration in real time and it is difficult to prevent the occurrence of eutrophication.

To solve these issues, a microfluidic chip with a novel pretreatment process is explored in this work, towards the goal of simpler TP analysis system design. To prevent the eutrophication, the concentration of TP in aquatic ecosystem must be monitored, quickly. Separating phosphate (PO_4_^3−^) from various compounds containing un-pretreated phosphorus is need in order to monitor the concentration of TP in aquatic ecosystem. This process is called TP pretreatment process.

Phosphate (PO_4_^3−^) separated by pretreatment is colorized blue by a colorizing agent, which is a mixture of ammonium molybdate ((NH_4_)_2_MoO_4_) and ascorbic acid, and the concentration of phosphorus contained in various compounds (analyte) is analyzed by measuring the absorbance of the colorized analyte. As mentioned above, the TP pretreatment process that separates the phosphate from phosphorus, which is combined with the various organic/inorganic substances, is most important in TP analysis process, and the analyte to be estimated is decomposed by utilizing a conventional pretreatment equipment called an autoclave. The conventional TP pretreatment process utilizing autoclave is conducted under harsh conditions such as a temperature higher than 120 °C, pressure of more than 1.1 kg·cm^−2^. Therefore, a conventional pretreatment equipment (autoclave) is mainly used in laboratory despite its bulky size, high cost, and its harsh pretreatment conditions such as high temperature and pressure [32,33,34,35,36,37,38,39]. It is especially difficult to minimize TP analysis or pretreatment system because of these harsh pretreatment conditions. Therefore, it is necessary to develop the novel TP pretreatment method, which is without harsh pretreatment conditions, and enables TP pretreatment process on a tiny chip.

In this study, a thermally enhanced photocatalyst-based TP pretreatment microfluidic chip is proposed and characterized by utilizing lab-on-a-chip (LOC) and micro-electro-mechanical systems (MEMS) technologies. The photocatalytic reaction is a method of decomposing various contaminants by utilizing a strong oxidative power of hydroxyl radical, which is generated by illuminating ultra-violet (UV) ray to catalyst. In previous work, we suggested a TP analysis chip with a photocatalytic reaction for developing a portable TP analysis equipment. It has many advantages such as small size, low cost, and small-volume consumption of analytes, but it takes a long time (about 20~30 min) to analyze TP [39]. To solve these weaknesses, a moderate thermal energy is added to the pretreatment part with photocatalytic reaction in order to improve pretreatment efficiency in this work. Because the proposed thermally enhanced photocatalytic reaction-based TP pretreatment microfluidic chip can operate under standard atmospheric conditions under a smaller footprint towards portability, it offers the greater freedom in device design and fabrication and is appropriate for developing a portable TP analysis system.

Fundamentally, a photocatalytic reaction is one where a chemical reaction is accelerated due to the presence of a catalyst. A photocatalytic reaction produces electron–hole pairs, which generate free radicals (hydroxyl radical in this study), when the photocatalyst absorbs ultra-violet (UV) radiation from sunlight or an artificial light source. In this paper, titanium dioxide (TiO_2_) was used as the photocatalyst, and a UV light-emitting lamp with a wavelength of 365 nm was used as the light source [39,40,41,42,43,44,45,46,47,48,49]. In addition, a micro-heater was also installed in the proposed pretreatment channel with a photocatalyst for improving the efficiency of the photocatalytic reaction. In order for a reaction between molecules to occur, they must exist as close to each other as infinity, and each molecule must have energy greater than the energy required for the reaction (activation energy, *Ea*). According to the Maxwell–Boltzmann distribution, as the temperature increases, the number of molecules with energy above the activation energy increases. Therefore, the efficiency of the photocatalytic reaction is increased by applying appropriate thermal energy to the photocatalytic reaction. Since the proposed pretreatment process is implemented with a thermally enhanced photocatalytic reaction, an excellent TP pretreatment performance is expected, although harsh pretreatment conditions such as high temperature, pressure, and a long time for analysis is not applied to the micro channel. Once the UV light illuminates the photocatalytic surface, phosphate ions (PO_4_^3−^) are separated from a variety of compounds containing un-pretreated phosphorus flowing in the channel. The proposed microfluidic chip for TP pretreatment contains two constituent components on a tiny single-chip: A mixing channel and a pretreatment channel with thermally enhanced photocatalyst. Thus, it is a potential portable candidate for minimizing the conventional TP analysis system, without time and location constraints.

## 2. Principle of TP Analysis Utilizing a Thermally Enhanced Photocatalyst

**Figure 1:**
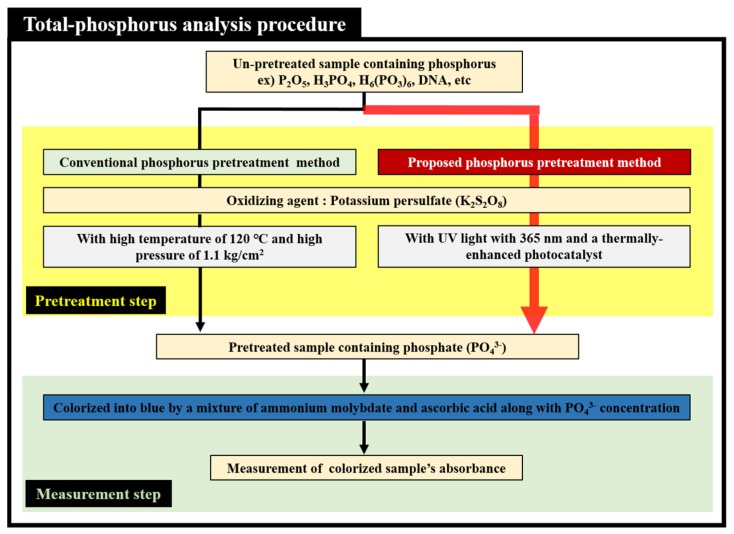
Principle of total-phosphorus (TP) analysis procedure employing thermally enhanced photocatalytic reaction.

Figure 1 shows the TP analysis procedure, consisting mainly of the pretreatment and the measurement steps. Before the concentration of TP is measured, phosphate ions (PO_4_^3−^) must be separated from various compounds containing un-pretreated phosphorus. In TP analysis procedure, the pretreatment step is very important because TP concentration cannot be measured exactly if phosphate ion (PO_4_^3−^) is not separated from various compounds containing un-pretreated phosphorus perfectly. To separate the phosphate ions (PO_4_^3−^) from compounds containing un-pretreated phosphorus, the analyte used in this study must undergo a pretreatment process that involves mixing with potassium persulfate (K_2_S_2_O_8_), and either applying high temperatures and pressures, or photocatalytic reaction by irradiating UV light. When high thermal or UV energy is applied to an analyte mixed with potassium persulfate, hydroxyl radicals with a strong oxidative power are produced. The hydroxyl radicals generated in the pretreatment process play the role of a strong oxidizing agent for decomposing various compounds containing un-pretreated phosphorus. After this process, the hydroxyl radicals separate the phosphate ions (PO_4_^3−^) from a variety of compounds containing un-pretreated phosphorus. A colorizing agent containing a mixture of ammonium molybdate ((NH_4_)_2_MoO_4_) and ascorbic acid is then added to the analyte containing the separated (or pretreated) phosphate ions (PO_4_^3−^). As a result, the color of the analyte turns blue, and its absorbance is optically measured at a wavelength of 880 nm or at a desired wavelength. Our study used a thermally enhanced photocatalytic reaction-based pretreatment process that was more appropriate for a miniaturized TP analysis system than the conventional pretreatment process involving harsh pretreatment conditions such as high temperatures and pressures. To improve the pretreatment efficiency, a micro-heater was also applied to the proposed TP pretreatment microfluidic chip with photocatalyst in order to supply a moderate thermal energy. The proposed microfluidic chip used TiO_2_ as photocatalyst by fabricating them in the pretreatment channel and fabricated photocatalyst (TiO_2_) accelerated chemical reactions without consumed as a reactant and being produced as contaminants (it only produces H_2_O and CO_2_ as by-products). The strong oxidizing effect of the TiO_2_ during UV irradiation lowered the activation energy required for the decomposition of organic and inorganic compounds [39,50,51,52].

## 3. Design and Fabrication of the Proposed TP Pretreatment Microfluidic Chip

**Figure 2:**
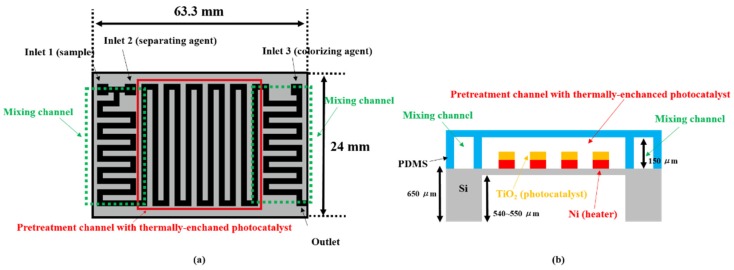
Schematic of the proposed TP pretreatment microfluidic chip. (**a**) Top view, (**b**) side view.

The proposed thermally enhanced photocatalytic reaction-based TP pretreatment microfluidic chip for minimizing TP analysis system has two constituent components: A mixing channel and a pretreatment channel. A schematic illustration of the designed TP pretreatment microfluidic chip is shown in Figure 2a,b.

The designed mixing channel is used for mixing the analyte containing un-pretreated phosphorus and potassium sulfate (K_2_S_2_O_8_) as pretreatment agent prior to the pretreatment process. Then, another mixing channel with the same structure is designed for mixing the pretreated analyte containing phosphate ion (PO_4_^3−^) and a colorizing agent that is a mixture of ammonium molybdate ((NH_4_)_2_MoO_4_) and ascorbic acid. The size of the proposed microfluidic chip is about 63.3 mm × 24 mm. Micro-mixer or mixing channel is very important to develop a microfluidic chip with high performance. There are various researchers focusing on improvement of the mixing performance in microfluidic chip. Methods for improving the mixing performance can be divided into passive and active mixing method. Active mixing methods manipulate and mix fluid quickly through various external forces or actuators or equipment, such as piezoelectric, thermal, acoustic, and magnetic force. Although active mixing methods utilizing external force or an actuator improve the mixing performance, they require complex structure and additional equipment or materials. Furthermore, they often suffer from problems associated with heat transfer, bubble formation, difficulties in design and fabrication, and complex structure. In contrast, passive mixing methods have the ability to improve the mixing performance without the requirement of external force or additional equipment. In general, passive mixing methods interrupt the flow of fluids and mix fluids by fabricating obstacles in the flow line and micro-channel with various shapes. Passive mixing method can be easily realized by utilizing MEMS technology and offers the greater freedom in microfluidic chip design and fabrication because it is not need external force or additional equipment. In this paper, the mixing channels were designed with a curvature structure in the flow line to achieve more effective mixing of the injected analyte with the separating and colorizing agents. As mentioned above, this method is usually called passive mixing method, which uses obstacles or curvature structures in the flow line. Although there is another method that can mix fluids in a short time (called active mixing method), it is difficult to fabricate and maintain a system that can perform this task at the microscale. Therefore, the passive mixing method (mixing channel) containing curvature structures in the flow line was adopted by considering difficulties and cost in design and fabrication step and the mixing performance [53,54,55,56,57].

The width and height of the mixing channel used in this study were 1000 µm and 150 µm, respectively. Without a complicated structure or fabrication process, the proposed mixing channel with several curvature structures enabled the effective mixing of the analyte and agents used in this study.

The pretreatment process that separates phosphate from various compounds containing un-pretreated phosphorus is an essential step in TP analysis. As previously mentioned, pretreatment processes for TP analysis can be roughly classified into two types: (1) The application of high heat and pressure and (2) photocatalytic methods using UV light. In the conventional pretreatment method, the analyte containing the un-pretreated phosphorus, potassium sulfate (K_2_S_2_O_8_), is heated to 120 °C for at least 30 min. In the pretreatment process with microfluidic chip, it would be necessary to pressurize the channel to 1.1 kg·cm^−2^ in order to increase the boiling point of the analyte and agent. However, these harsh pretreatment conditions cannot be realized in a microfluidic chip because of its weak structures physically. On the other hand, a pretreatment process utilizing photocatalytic reaction was performed by irradiating UV light to the fabricated TiO_2_ in microfluidic chip. Hydroxyl radicals with a strong oxidative power were produced, and phosphate ions (PO_4_^3−^) were separated from various compounds containing un-pretreated phosphorus when UV light illuminated the TiO_2_ photocatalytic surface. In addition, a moderate thermal energy through a micro-heater is applied to the proposed pretreatment channel to improve the photocatalytic efficiency. The proposed pretreatment process in this study has many advantages compared to the conventional heating method, including the absence of high pretreatment temperature (thermal energy) and pressurization, low cost, and greater freedom in device design and fabrication.

**Figure 3:**
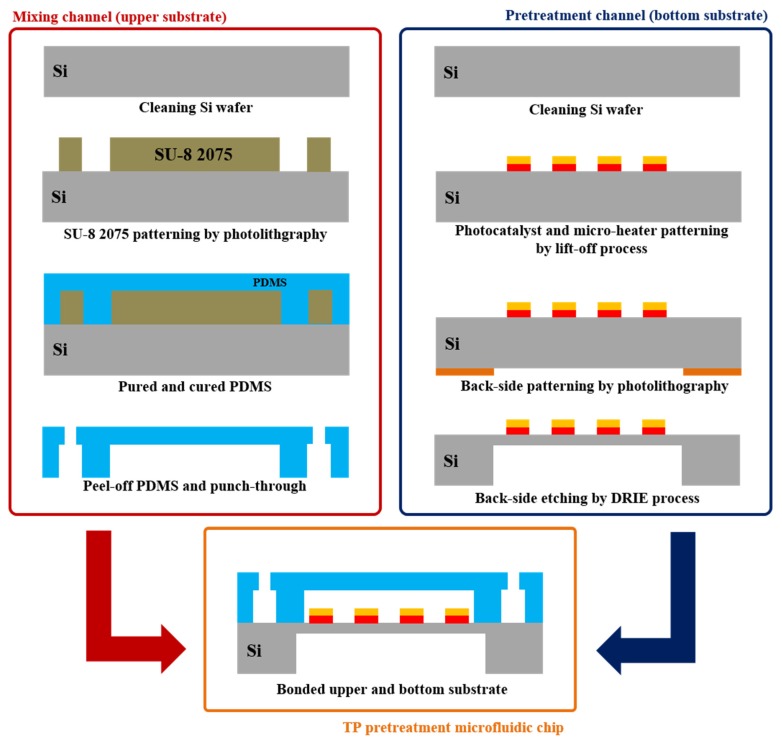
Fabrication process of the proposed TP pretreatment microfluidic chip.

The proposed TP pretreatment microfluidic chip with thermally enhanced photocatalytic reaction was fabricated using LOC and MEMS technologies, as shown in Figure 3.

The purpose of mixing channel in microfluidic chip is to mix the analyte (containing un-pretreated phosphorus), the separating agent (potassium sulfate, K_2_S_2_O_8_), and the colorizing agent. The separating agent helps to separate the phosphorus from various compounds while the colorizing agent turns blue with changing concentrations of phosphorus. First, the silicon (Si) wafer is cleaned with acetone and methanol to fabricate the mixing channel. The SU-8 2075 negative PR is then patterned on the Si wafer through a photolithography process. The width and height of the patterned SU-8 2075 were 1000 μm and 150 μm, respectively. Because the patterned SU-8 2075 is used as a mold, the width and height of the fabricated SU-8 2075 determine the width and height of the mixing channel and pretreatment channel. Un-cured PDMS is poured on the patterned SU-8 2075 and, PDMS curing process is performed by utilizing oven. Cured PDMS is peeled-off from the fabricated mold with patterned SU-8 2075, and the inlet and outlet are fabricated through punch-through process. Second, the Si wafer is also cleaned with acetone and methanol to fabricate the pretreatment channel with photocatalyst and a micro-heater. Titanium (Ti) with thickness of 20 nm and nickel (Ni) with thickness of 60 nm are deposited by the e-beam evaporation process. The deposited Ti/Ni layer is used as a micro-heater. The fabricated micro-heater generates a moderate thermal energy and it helps to enhance the efficiency of pretreatment with photocatalyst. The titanium dioxide layer (TiO_2_) used as photocatalyst is then deposited on the fabricated micro-heater through sputtering process. Un-pretreated phosphorus contained in various compounds is separated (or pretreated) by the deposited photocatalyst without high temperature and pressure. After a micro-heater and photocatalyst fabrication processes, the reverse side of the Si wafer is etched using the deep Si etch (DRIE) process. The membrane fabricated by the DRIE process serves as a thermal isolation structure. Finally, the fabricated mixing part utilizing cured PDMS and pretreatment part are bonded.

## 4. Results and Discussion

**Figure 4:**
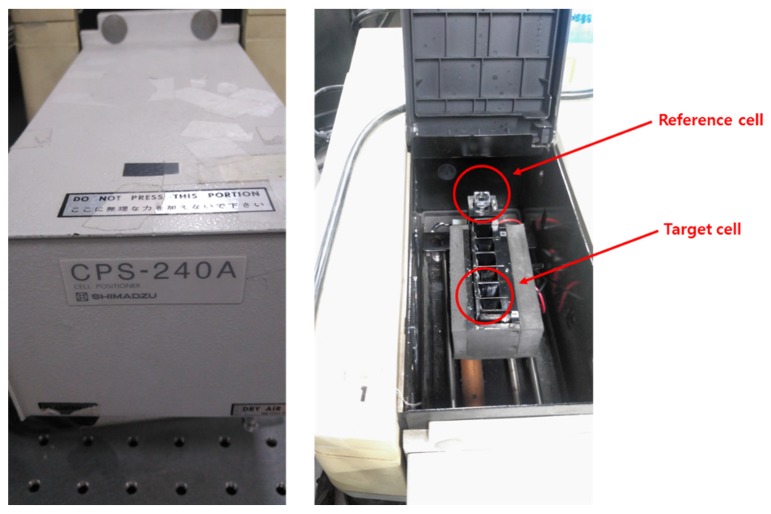
UV-Vis spectrometer for measuring colorized analyte’s absorbance.

Before TP pretreatment using the proposed microfluidic chip is performed, the absorbance of the prepared phosphate standard solution was measured using UV-Vis spectrometer (model: CPS-240A, Shimadzu Inc., Kyoto, Japan) to study the relationship between the phosphate concentration and its absorbance, as shown in Figure 4.

**Figure 5:**
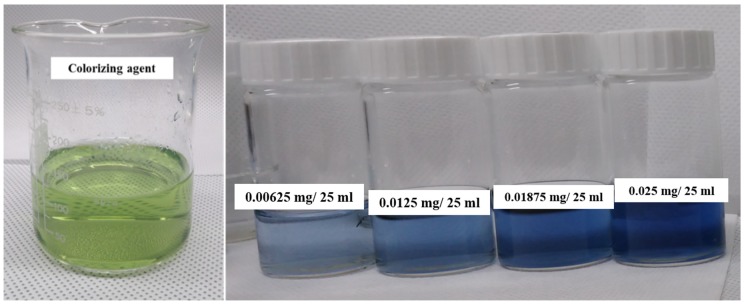
Images of the prepared colorizing agent and the turned blue standard solutions along with various phosphate concentrations.

**Figure 6:**
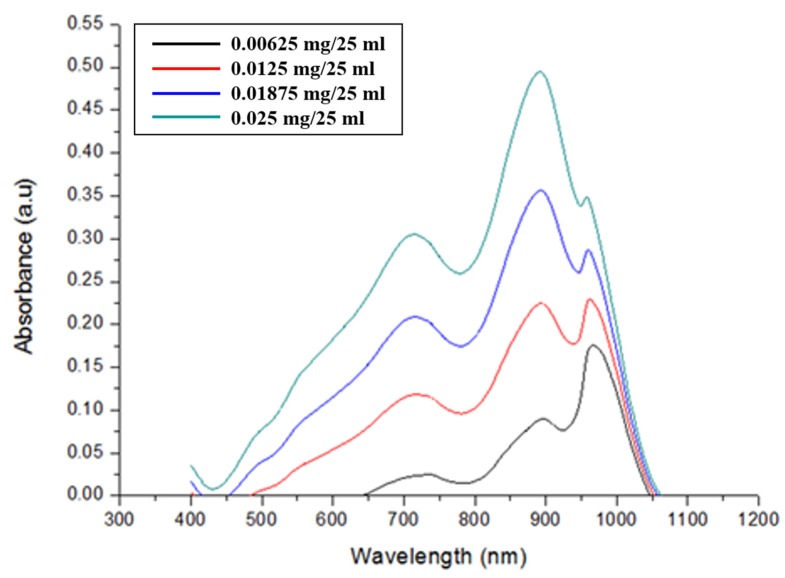
Measured absorbance of standard phosphate solution along with various phosphate concentrations.

**Figure 7:**
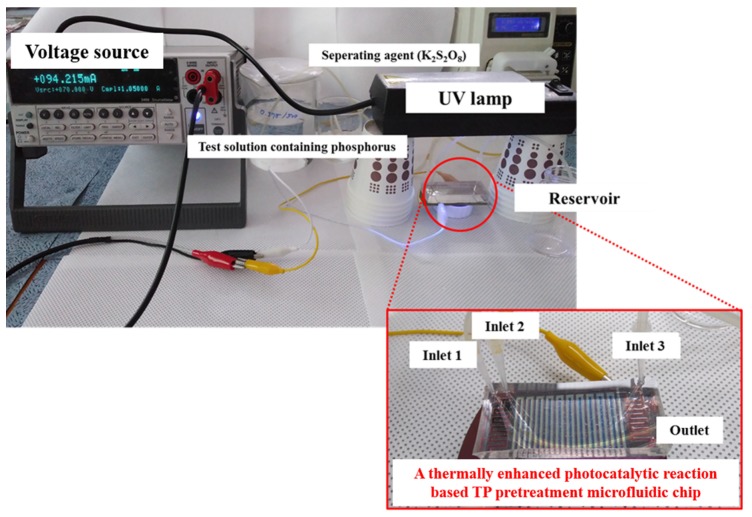
Experimental setup to estimate the proposed TP pretreatment microfluidic chip.

**Figure 8:**
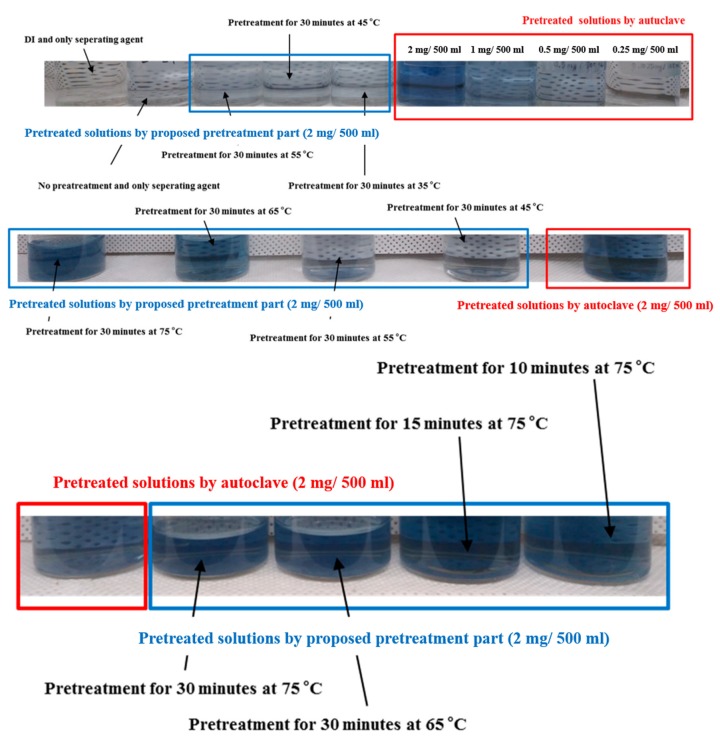
Comparison of the pretreatment performance between the proposed TP pretreatment microfluidic chip and the conventional pretreatment equipment.

The prepared colorizing agent and standard phosphate solutions were mixed, and the mixed solutions turned blue in accordance with the phosphate concentration, as shown in Figure 5. The absorbance of the colorized solutions was measured using the UV-Vis spectrometer. Figure 6 shows that the measured absorbance increases with increase in the concentration of the phosphate and their maximum absorption wavelength lies at 880 nm. Other absorption peaks were also measured at wavelengths of 710 and 975 nm. This means that concentration of phosphates can be measured by estimating the absorbance of the colorized phosphate solution. Based on these experimental results, the proposed TP pretreatment microfluidic chip based on thermally enhanced photocatalytic reaction is fabricated and characterized in order to minimize conventional TP analysis system. Figure 7 shows the experimental setup for estimating the proposed and fabricated TP pretreatment microfluidic chip using the thermally enhanced photocatalytic reaction. The prepared test solution containing un-pretreated phosphorus (2 mg/500 mL) and the separating agent (K_2_S_2_O_8_) were injected through inlet 1 and 2, respectively. Then, a mixture of test solution and separating agent flows in pretreatment channel. Voltage source (2400 Source meter, Keithley, Cleveland, OH, USA) was used to apply voltage to a micro-heater in the proposed pretreatment channel. The temperature for the fabricated pretreatment channel was modulated by changing the applied voltage. Through the characteristics of the fabricated heater, the appropriate temperature for the pretreatment channel was achieved by applying the proper voltage values to a micro-heater. When different voltages were applied to the fabricated micro-heater, the temperatures achieved in the pretreatment channel were: 35 °C (45 V), 45 °C (56 V), 55 °C (67 V), 65 °C (77 V), and 75 °C (86 V), respectively. The expected temperature of a micro-heater could be estimated by utilizing infrared (IR) thermometer with non-contact method (model: FLK-62MAX, manufacturer: FLUKE, Everett, DC, USA). The time taken for the pretreatment can be changed by modulating the flow rate of the micro pump (Longer Pump, BT100-1F). The test solutions are pretreated under different pretreatment temperatures and times, and the pretreated test solutions turned blue when mixed with a colorizing agent injected through inlet 3, as shown in Figure 8.

**Figure 9:**
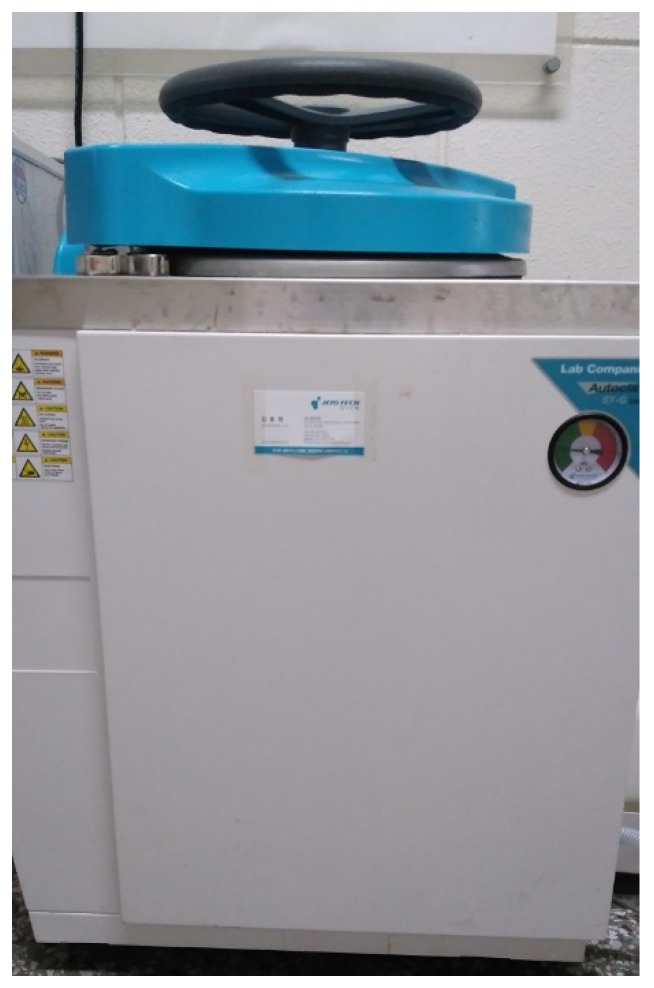
Image of the conventional pretreatment equipment (Autoclave).

**Figure 10:**
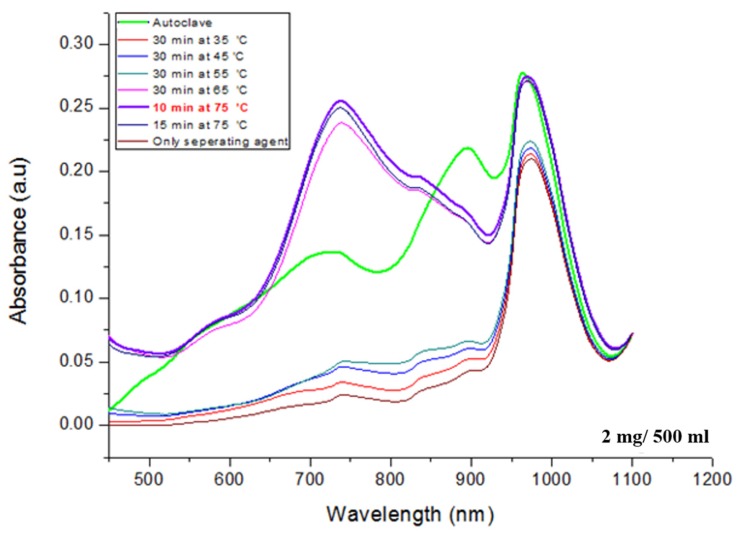
Measured absorbance of the pretreated test solution (2 mg/500 mL) using the thermally enhanced photocatalytic reaction based microfluidic chip and autoclave.

To estimate the performance of the proposed TP pretreatment microfluidic chip using thermally enhanced photocatalysts, a test solution with 2 mg/500 mL of phosphorus concentration was also pretreated using the conventional equipment (autoclave) as reference. Figure 9 shows the image of the conventional pretreatment equipment, called autoclave. The pretreatment process using autoclave was conducted for 30 min at 120 °C. The test solution with 2 mg/500 mL of phosphorus concentration was also pretreated using the proposed TP pretreatment microfluidic chip at the same time. All experiments with the proposed TP pretreatment microfluidic chip were conducted by changing the pretreatment temperature and times. The experimental results show that the pretreated test solutions using the proposed TP pretreatment microfluidic chip turned deep blue as the pretreatment temperature and time increased. The absorbance of the colorized test solutions was measured by UV-vis spectrometer, as shown in Figure 10.

In Figure 10, the measured absorbance of the pretreated test solution (2 mg/500 mL) for 10 min at 75 °C showed similar characteristics to the conventional pretreatment equipment. Experimental results show that measured absorbance of the pretreated test solution is increased by increasing pretreatment temperature, and it is increased between 55 °C and 65 °C, dramatically. It means that the number of molecules with energy above the activation energy are rapidly increased at specific temperature between 55 °C and 65 °C while they are saturated at temperatures above 75 °C.

Generally, the pretreatment performance is improved by increasing the pretreatment temperature but if the pretreatment process takes too long (30 min in this experiment) at 75 °C, the performance would be degraded, as shown in Figure 10. It is believed that if the pretreatment process at high temperatures takes too long, the phosphorus contained in solution are decomposed and, it is actively recombined with various substances in parallel. From the experimental results, it is confirmed that the optimum time and temperature for the proposed TP pretreatment microfluidic chip are 10 min and 75 °C, respectively. The performance of the proposed TP pretreatment microfluidic chip under these conditions correspond to that of the conventional pretreatment equipment.

**Figure 11:**
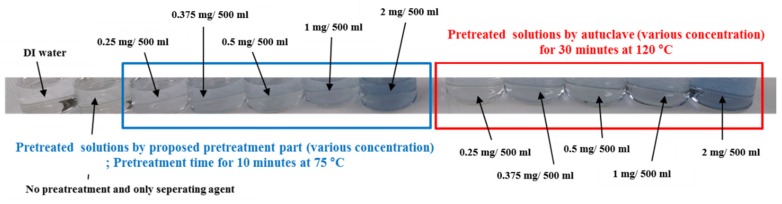
Comparison of the pretreatment performance between the proposed method and the conventional method at different concentrations of phosphorus.

**Figure 12:**
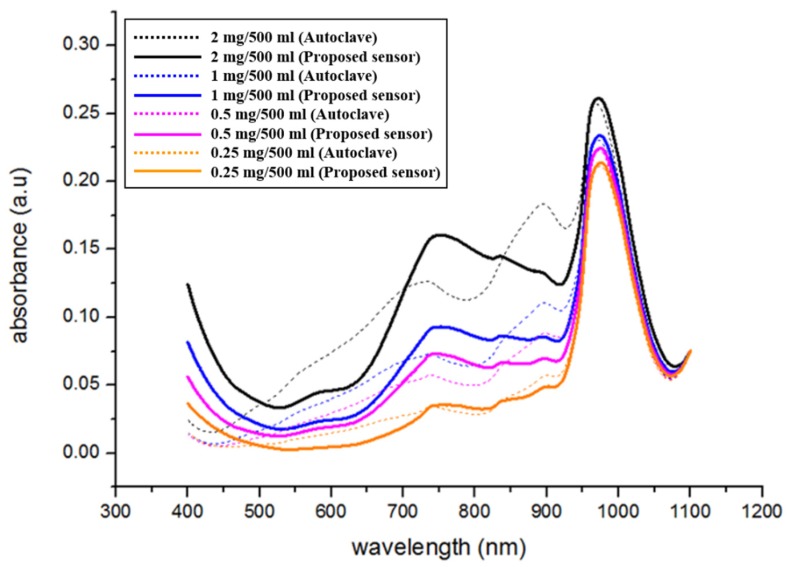
Comparison of the pretreatment performance between the optimized pretreatment method and the conventional method at different phosphorus concentrations.

Finally, the test solutions with various phosphorus concentration were pretreated using the proposed microfluidic chip with optimized pretreatment condition and conventional pretreatment equipment, and their absorbance was measured by using the UV-Vis spectrometer. Figure 11 shows the pretreated test solutions using the proposed microfluidic chip and conventional pretreatment equipment. Similar to the previous experiments, the color of pretreated test solutions turned blue and grew deeper with increasing concentration of phosphorus. In addition, the color changes in the pretreated test solutions under different phosphorus concentrations were similar for both the optimized pretreatment (10 min at 75 °C) and conventional pretreatment methods (30 min at 120 °C). To estimate the optimized microfluidic chip and conventional pretreatment equipment quantitatively, the absorbances of the test solutions were measured. The measured absorbance of pretreated test solutions using the proposed microfluidic chip with optimized condition was also similar to that of the pretreated test solutions using the conventional pretreatment equipment, at different concentrations of phosphorus, as shown in Figure 12.

**Figure 13:**
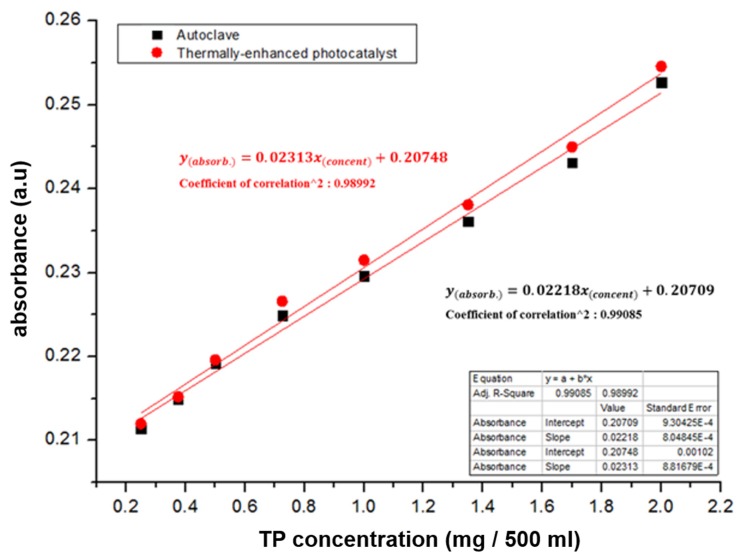
Comparison of the relationship between phosphorus concentrations and measured absorbance at a wavelength of 975 nm.

The measured absorbance peak lies at a wavelength of 710 nm and 975 nm. Employing the absorbance at 975 nm is appropriate for measuring the differences in absorbances under different concentrations of phosphorus considering measured differences in absorbance and its linearity under different concentrations of phosphorus. The measured absorbance at 975 nm increases with increasing phosphorus concentrations. Figure 13 shows a comparison of the measured absorbance with variations in phosphorus concentration using the proposed microfluidic chip and using the conventional equipment. Absorbance along with variations in phosphorus concentration was measured at 975 nm. The measured absorbance along with variations in phosphorus concentration using the proposed microfluidic chip has similar characteristics as that of the conventional equipment. The equations between the measured absorbance as a function of variations in phosphorus concentrations was derived as follows:

$$y_{absorb.}=0.02312x_{concent}+0.20748\left(by\;thermally-enhanced\;photocatalyst\right)$$

$$y_{absorb.}=0.02218x_{concent}+0.20709\left(by\;autoclave\right).$$

Therefore, an unknown analyte’s phosphorus concentration could be expected by using the derived relationship between the measured absorbance along with various phosphorus concentrations utilizing the proposed microfluidic chip.

The results of the experiment performed show that the proposed and optimized TP pretreatment microfluidic chip with thermally enhanced photocatalytic reaction could replace the conventional pretreatment equipment, which is very bulky and expensive. The proposed and optimized microfluidic chip for phosphorus pretreatment offers advantages such as small size, short pretreatment time, and is inexpensive compared to the conventional pretreatment system. This is a critical factor for developing portable TP analysis systems.

## 5. Conclusions

In this study, a TP pretreatment microfluidic chip with thermally enhanced photocatalytic reaction was proposed and characterized to develop a portable TP analysis system. Phosphorus exists as phosphate (PO_4_^3−^) or other compounds and is an important nutrient for living organisms. Increasing the concentration of phosphorus leads to eutrophication of the aquatic ecosystem. Therefore, there is a growing need to monitor phosphorus concentrations in real time to prevent eutrophication. TP analysis consists mainly of the two steps: Pretreatment and measurement. The pretreatment step is used to separate the phosphate (PO_4_^3−^) from various compounds containing phosphorus while the measurement step is used to measure the phosphorus concentration using the measured absorbance of the phosphate that is colorized blue. The pretreatment step is very critical in TP analysis because virtually entire phosphates (PO_4_^3−^) in aquatic systems exist as compounds. Autoclave is the conventional equipment for TP pretreatment. It is very bulky, expensive, and operates under the following conditions: 30 min pretreatment time, 120 °C of pretreatment temperature, and 1.1 kg/cm^2^ pretreatment pressure. To monitor phosphorus concentration in real time, it is important to make the TP analysis system portable. A microfluidic chip using LOC and MEMS technologies is an appropriate candidate for this application. However, it is difficult to fabricate a microfluidic chip that operates effectively under high temperature and pressure. To solve these problems, a pretreatment method using a photocatalyst (TiO_2_) is applied to the proposed microfluidic chip, and moderate thermal energy is also added to the photocatalytic reaction to improve the efficiency of the pretreatment. The photocatalytic reaction was accelerated by the photocatalyst and the hydroxyl radicals generated by the photocatalytic reaction decomposite phosphorus in different compounds into phosphates. Because of the high oxidizing strength of the hydroxyl radicals, the pretreatment process can be conducted effectively without high temperature and pressure. Therefore, it leads to the miniaturization of the TP analysis system. In previous work, we suggested a TP analysis chip with photocatalytic reaction for developing a portable TP analysis equipment. Despite the proposed microfluidic chip’s many advantages such as small size, low cost, and small-volume consumption of analytes, the proposed microfluidic chip in previous work takes a long time (about 20~30 min) to analyze TP [53]. To improve this weakness, a microfluidic chip with a photocatalyst and micro-heater was fabricated and characterized in this study. The proposed microfluidic chip offers ease of design and fabrication because high temperatures and pressures were unnecessary. It also enables the development of portable TP analysis systems.

First, the microfluidic chip with the photocatalyst (TiO_2_) and micro-heater were fabricated using MEMS and LOC technologies. Test solutions containing phosphorus were pretreated by utilizing the fabricated TP pretreatment microfluidic chip under the various pretreatment conditions such as temperature and time. After the test solution containing phosphorus was pretreated using the fabricated microfluidic chip, the pretreated test sample was colorized blue in response to varying phosphorus concentrations. Next, the concentration of the phosphorus was quantitatively measured by estimating the absorbance of the colorized test sample. The measured absorbance of the colorized test sample was increased by increasing phosphorus concentrations. The proposed TP pretreatment microfluidic chip with thermally enhanced photocatalyst was optimized compared to the conventional pretreatment equipment (autoclave). The optimum pretreatment conditions using the proposed chip were 10 min of pretreatment time and 75 °C as pretreatment temperature. The experimental results show that the efficiency of the pretreatment with photocatalyst improves as the pretreatment temperature increases. This is because a number of molecules with energies greater than the activation energy were increased, following the Maxwell–Boltzmann distribution. However, the efficiency drops if pretreatment process is conducted for too long at 75 °C. This is because there is enough time for the recombination of the pretreated phosphate. The optimized pretreatment process using the proposed microfluidic chip showed similar performance to the conventional pretreatment method, even with shorter pretreatment time compared to the TP analysis microfluidic chip developed in previous work [53]. The shorter pretreatment time and small size are advantages that can be used to develop a portable TP analysis system. Therefore, the proposed TP pretreatment microfluidic chip based on thermally enhanced photocatalytic reaction in this study will be utilized to develop a portable TP analysis system, which integrates the pretreatment, mixing, and measurement parts on a tiny device. In future works, the measurement part with multi-reflective structure will be developed and be combined with the proposed TP pretreatment microfluidic chip based on thermally enhanced photocatalytic reaction.
